# Impact of liver PGC‐1α on exercise and exercise training‐induced regulation of hepatic autophagy and mitophagy in mice on HFF

**DOI:** 10.14814/phy2.13731

**Published:** 2018-07-01

**Authors:** Maja M. Dethlefsen, Caroline M. Kristensen, Anna S. Tøndering, Signe B. Lassen, Stine Ringholm, Henriette Pilegaard

**Affiliations:** ^1^ Department of Biology Section for Cell Biology and Physiology University of Copenhagen Kobenhavn Denmark

**Keywords:** Acute exercise, autophagy, exercise training, high‐fat high‐fructose, liver, Liver PGC‐1α KO, mitophagy

## Abstract

Hepatic autophagy has been shown to be regulated by acute exercise and exercise training. Moreover, high‐fat diet‐induced steatosis has been reported to be associated with impaired hepatic autophagy. In addition, autophagy has been shown to be regulated by acute exercise and exercise training in a PGC‐1α dependent manner in skeletal muscle. The aim of this study was to test the hypotheses that high‐fat high‐fructose (HFF) diet changes hepatic autophagy and mitophagy, that exercise training can restore this through a PGC‐1α‐mediated mechanism, and that acute exercise regulates autophagy and mitophagy in the liver. Liver samples were obtained from liver‐specific PGC‐1α KO mice and their littermate Lox/Lox mice fed a HFF diet or a control diet for 13 weeks. The HFF mice were either exercise trained (ExT) on a treadmill the final 5 weeks or remained sedentary (UT). In addition, half of each group performed at the end of the intervention an acute 1 h exercise bout. HFF resulted in increased hepatic BNIP3 dimer and Parkin protein, while exercise training increased BNIP3 total protein without affecting the elevated BNIP3 dimer protein. In addition, exercise training reversed a HFF‐induced increase in hepatic LC3II/LC3I protein ratio, as well as a decreased PGC‐1α mRNA level. Acute exercise increased hepatic PGC‐1α mRNA in HFF UT mice only. In conclusion, this indicates that exercise training in part reverses a HFF‐induced increase in hepatic autophagy and capacity for mitophagy in a PGC‐1α‐independent manner. Moreover, HFF may blunt acute exercise‐induced regulation of hepatic autophagy.

## Introduction

The liver is an essential organ with key functions in whole‐body glucose and lipid metabolism. Previous studies have shown that high‐fat diet is associated with hepatic triglyceride accumulation and disturbed liver metabolism (Delgado *et al*. 2009, Koonen *et al*. 2007, Schults *et al*. 2012). Several processes have been suggested to be affected, including recent indications that high‐fat diet changes the regulation of autophagy in the liver.

Autophagy is a catabolic process targeting damaged proteins and cell organelles for lysosomal degradation, thereby supporting cellular survival and maintenance of homeostasis (Klionsky *et al*. 2016). The energy sensors mammalian target of rapamycin (mTOR) and AMP‐activated protein kinase (AMPK) are important checkpoints in the regulation of autophagy, exerting inhibitory (ULK^Ser757^) or activating (ULK^Ser317^) phosphorylation of Unc‐51‐like kinase 1 (ULK1), respectively (Komatsu 2012). Moreover, initiation of a phagophore lipid bilayer membrane is controlled by an ULK1 complex, while Beclin1 is involved in recruiting lipid for elongation of the phagophore membrane (Komatsu 2012, Lavallard & Gual 2014). The adaptor protein sequestosome‐1 (p62) selectively binds and transports ubiquitinated cellular components and through direct interaction with microtubule‐associated proteins 1A/1B light chain 3B (LC3), the cargo is incorporated along with p62 into the autophagosome. The lipidated form of LC3I (LC3II) completes the enclosure of the phagophore, and is therefore used as a measure of autophagosome number (Komatsu 2012). Furthermore, changes in the LC3II/LC3I protein ratio in combination with p62 protein are often used as indicators of changes in autophagy (Komatsu 2012, Lavallard & Gual 2014). In addition, mitophagy is a specialized form of autophagy targeting damaged mitochondria (Lemasters 2005). One mitophagy pathway is through E3 ubiquitin‐protein ligase parkin (Parkin), which is recruited to damaged mitochondria marking these for degradation through p62 binding. Another pathway directly targets damaged mitochondria for degradation by BCL2/adenovirus E1B 19 kDa protein‐interacting protein 3 (BNIP3) binding to LC3II to induce mitophagy (Youle & Narendra 2011).

The process of autophagosomal fusion with lysosomes has been reported to be sensitive to changes in membrane‐lipid composition, and the capacity of the fusion process is decreased in livers from mice fed a high‐fat diet (HFD) (Koga *et al*. 2010) indicating changed autophagy with HFD feeding. In addition, previous studies have shown that HFD increased the LC3II/LC3I ratio and/or LC3II protein content (Gonzalez‐Rodriguez *et al*. 2014, Hsu *et al*. 2016, Tanaka *et al*. 2016, Wang *et al*. 2017a), increased AMPK phosphorylation (Hsu *et al*. 2016) as well as decreased phosphorylation of mTOR and the inhibitory site on ULK1 in mouse liver (Tanaka *et al*. 2016). This may suggest that HFD induces increased autophagy. On the other hand, other studies have reported that HFD results in decreased LC3II protein and/or AMPK phosphorylation (Barroso *et al*. 2011, Ghareghani *et al*. 2017, Liu *et al*. 2015, Rosa‐Caldwell *et al*. 2017, Wang *et al*. 2017b) as well as increased hepatic p62 protein (Ghareghani *et al*. 2017, Gonzalez‐Rodriguez *et al*. 2014, Liu *et al*. 2015, Tanaka *et al*. 2016, Wang *et al*. 2017a) indicating that HFD is associated with inhibition of hepatic autophagy. Together this underlines that the impact of HFD on the regulation of hepatic autophagy remains to be fully resolved.

Exercise training has been reported to exert numerous beneficial effects on hepatic metabolism in rodents on HFD. Thus, exercise training has been shown to reduce or even reverse HFD‐induced hepatic triglyceride (TG) accumulation (Alex *et al*. 2015, Wang *et al*. 2017a) as well as inflammation and insulin resistance (Kawanishi *et al*. 2012). In addition, one study in mice has reported that exercise training prevented the HFD‐induced changes in AMPK and mTOR phosphorylation, LC3I, LC3II and p62 protein in the liver, although only representative blots were provided (Ghareghani *et al*. 2017). Moreover, voluntary wheel running has been reported to partially restore diet‐induced mitochondrial quality impairment in mouse skeletal muscle (Greene *et al*. 2015). However, whether exercise training performed after several weeks of HFD can restore hepatic autophagy and mitophagy regulation is not known.

Previous studies have shown that a single exercise bout regulates autophagy in skeletal muscle (Halling *et al*. 2016, Vainshtein *et al*. 2015) and in liver (He *et al*. 2012), but whether long‐term HFD intake and concomitant hepatic TG accumulation affects the exercise‐induced autophagy regulation in the liver remains to be determined.

Several factors may be involved in mediating exercise training‐induced adaptations in the liver. The transcriptional coactivator Peroxisome proliferator‐activated receptor gamma coactivator 1‐alpha (PGC‐1α) has been shown to be mandatory for exercise training‐induced adaptations in oxidative markers, but not gluconeogenic proteins in the liver of young mice on regular chow diet (Haase *et al*. 2011). Furthermore, PGC‐1α has been demonstrated to be required for the exercise training‐induced increase in LC3II in skeletal muscle and to be required for exercise training‐induced adaptations in LC3I (Brandt *et al*. 2018) and LC3II protein (Brandt *et al*. 2017). Moreover, PGC‐1α was required for an acute exercise‐mediated increase in LC3II protein content in skeletal muscle (Halling *et al*. 2016, Vainshtein *et al*. 2015) and has been shown to increase in mouse liver with an acute bout of exercise (Hoene *et al*. 2009). However, whether PGC‐1α influences basal and acute exercise‐induced hepatic autophagy regulation when on HFD and whether PGC‐1α is required for exercise training‐induced adaptations in hepatic autophagy when on HFD, remain to be resolved.

Therefore, the aim of this study was to test the hypotheses that (1) High‐fat high‐fructose (HFF) diet changes autophagy and mitophagy in the liver and exercise training performed after several weeks of HFF diet can restore hepatic autophagy regulation, (2) Hepatic autophagy is regulated by acute exercise in a PGC‐1α‐dependent manner and (3) PGC‐1α influences basal regulation of hepatic autophagy when on HFF and liver PGC‐1α is required for exercise training‐induced adaptations in hepatic autophagy and mitophagy when on HFF.

## Materials and Methods

### Mice

The present study used male C57BL/6N PGC‐1α liver‐specific knock out (LKO) mice and Lox/Lox littermate control mice. The mice were obtained by intercross breeding of mice homozygous for loxP flanked PGC‐1α alleles (Geng *et al*. 2010) and mice homozygous for floxed PGC‐1α and heterozygous for albumin‐cre (Postic & Magnuson 1999) as previously described (C. M. Kristensen, M. M. Dethlefsen, A. S. Tøndering, S. B. Lassen, J. N. Meldgaard, S. Ringholm, H. Pilegaard, unpublished data). Initially the genotypes were determined using PCR on gDNA isolated from an ear piece and determining the floxed PGC‐1α alleles and presence/absence of albumin‐cre. After euthanization, the genotypes were confirmed by determining PGC‐1α mRNA levels in the liver of all mice (Table 1). Mice were group‐housed until the beginning of the intervention period, with a 12:12 h light:dark cycle with ad libitum access to chow (Altromin no. 1324; Brogården, Lynge, Denmark) and water. Experiments were conducted in accordance with EU directive 2010/63/EU on protection of animals used for scientific purposes and approved by the Animal Experiment Inspectorate in Denmark (2013‐2934‐00911). In vivo data and analyses on liver samples from these mice have previously been reported (C. M. Kristensen, M. M. Dethlefsen, A. S. Tøndering, S. B. Lassen, J. N. Meldgaard, S. Ringholm, H. Pilegaard, unpublished data).

**Table 1:** PGC‐1α exon 3‐5 mRNA in liver from liver‐specific PGC‐1α knockout (LKO) and littermate control (Lox/Lox) mice fed a control diet (CON) or a high‐fat high‐fructose diet (HFF UT) for 13 weeks

Group		PGC‐1α exon 3‐5 mRNA	
		Lox/Lox	PGC‐1α LKO
CON UT	Sed	1.01 ± 0.1	0.01 ± 0.0
HFF UT	Sed	0.66 ± 0.1	0.01 ± 0.0
HFF UT	Ex	0.92 ± 0.1	ND
HFF ExT	Sed	0.87 ± 0.1	0.01 ± 0.0
HFF ExT	Ex	0.94 ± 0.1	0.01 ± 0.0

Half of the HFF mice performed treadmill exercise training the last 5 weeks (HFF ExT). By the end of the 13 weeks, the two groups were again divided into sedentary (HFF UT Sed and HFF ExT Sed) groups and groups performing a 1 h acute running bout (HFF UT Ex and HFF ExT Ex). Values are presented as means ± SE, *n *= 9–10. PGC‐1α exon 3–5 mRNA primers used are located within exon 3–5 which are deleted in PGC‐1α LKO mice.

### Experimental setup

Eight‐ to nine‐week‐old LKO and Lox/Lox littermate mice were randomly divided into two groups, receiving either a high‐fat high‐fructose (HFF) diet (D09100304: 20% protein, 40% carbohydrates and 40% fat) where 50% of the calories were derived from fructose (Research Diets, Inc., New Brunswick, NJ) or a matched control diet (CON) (D09100304: 20% protein, 70% carbohydrates and 10% fat) ad libitum (Research Diets, Inc.). Fructose was used to induce more severe liver steatosis as previously reported (Trevaskis *et al*. 2012). Body weight and food intake were registered weekly throughout the intervention period. In addition, all mice were MR scanned (Echo MRI, Echo Medical Systems) at intervention start, 8 weeks into the intervention and 2 days prior to euthanization.

After receiving the respective diet for 9 weeks, the HFF group was further divided into a HFF untrained (HFF UT) and a HFF exercise trained (HFF ExT) group. The HFF ExT group was adapted to the treadmill (TSE Systems GmbH, Bad Homburg, Germany) for 10 min 2 times per day for 4 days, before starting the exercise training protocol, consisting of 1 h of treadmill running (TSE Systems GmbH, Bad Homburg, Germany) at 15 m/min with 10^o^ incline, once a day, 6 days/week for 5 weeks.

By the end of the 14th week, the CON UT, HFF UT and HFF ExT groups were each divided into sedentary (CON UT, HFF UT, HFF ExT) and acute exercise groups (CON Ex, HFF UT Ex, HFF ExT Ex) resulting in 6 groups in total. The acute exercise bout consisted of 1 h of treadmill running (TSE Systems GmbH, Bad Homburg, Germany) at 15 m/min with 10^o^ incline and the mice were euthanized 2 h after the acute exercise running bout, together with the sedentary mice. The 2 h time point was chosen based on the previous findings that UPR markers were regulated at this time point (Kristensen *et al*. 2018). Exercise trained mice performed the last exercise bout 24 h prior to euthanization to prevent acute effects of exercise. All mice were euthanized by cervical dislocation at ~12 am–2 pm, trunk blood was quickly collected, and liver was removed and snap‐frozen in liquid nitrogen. Plasma was obtained by centrifugation of the blood at 2600 *g* and 4°C for 15 min. Liver and plasma samples were stored at −80°C until further analyses. To ensure homogeneity of the liver tissue, liver samples were crushed in liquid nitrogen before analyses.

### Analyses

#### RNA isolation and reverse transcription

Total RNA was isolated from crushed liver samples (~20 mg) by a modified guanidinium thiocyanate‐phenol‐chloroform extraction method (Chomczynski & Sacchi 1987) as previously described (Pilegaard *et al*. 2000), except that tissues were homogenized for 2 min at 30 sec−^1^ in a TissuelyserII (Qiagen, Germany). RNA concentration and purity were determined by spectrophotometry (Nanodrop 1000, Thermo Fischer Scientific). Reverse transcription was carried out on 3 *μ*g RNA using the Superscript II RNase H− and Oligo dT system (Invitrogen, Carlsbad, CA), as previously described (Pilegaard *et al*. 2000), and cDNA samples were diluted in nuclease‐free H_2_O.

### Real‐time PCR

mRNA content of PGC‐1α was measured using Real‐time PCR on a ABI‐7900 Sequence Detection System (Applied Biosystems, Forster City, CA). Primers and 5'‐6‐carboxyfluorescein (FAM) / 3'‐6‐carboxy‐N,N,N',N'‐tetramethylrhodamine (TAMRA) labeled Taqman probe were designed using Primer express 3.0 software (Applied Biosystems) and PGC‐1α forward primer (5' AGCCAAACCAACAACTTTATCTCTTC 3'), reverse primer (5' TTAAGGTTCGCTCAATAGTCTTGTTC 3') and Taqman probe (5' AGAGTCACCAAATGACCCCAAGGGTTCC 3') were obtained from TAG Copenhagen (Copenhagen, Denmark). Real‐time PCR was run in triplicates in a total reaction volume of 10 *μ*L using Universal Mastermix (Applied Biosystems). A standard curve, constructed from a dilution of a pooled portion of the cDNA samples, was run with the samples, and used to convert the cycle thresholds to a relative amount. The PGC‐1α mRNA content of each sample was normalized to total single stranded (ss) DNA content in the sample, determined with OliGreen reagent (Molecular Probes, Leiden, The Netherlands) as previously described (Lundby *et al*. 2005).

### Lysate preparation

Crushed liver tissue (~25 mg) was homogenized in ice‐cold buffer as previously described (Birk & Wojtaszewski 2006) using a TissuelyserII (Qiagen, Germany) for 2 min at 30 sec^−1^, followed by end over end rotation at 4°C for 1 h and subsequent centrifugation for 20 min at 16,000*g*. Total protein content was determined using the bicinchoninic acid method (Pierce Biotechnology Inc., Rockford, IL) and prepared in sample buffer containing sodium dodecyl sulfate (SDS) to a protein concentration of 2 μg *μ*L^−1^. Samples were boiled for 3 min at 96°C and analyzed by SDS‐PAGE and western blotting.

### SDS‐PAGE and western blotting

Protein content and phosphorylation of specific proteins were measured in the liver lysates by SDS‐PAGE loading equal amounts of protein in hand casted gels with appropriate acrylamide percentages (Tris‐HCl 8–15%). Thereafter, western blotting was performed using PVDF membranes (Immobilon‐P Transfer membranes, Millipore) and semi‐dry transfer, as previously described (Birk & Wojtaszewski 2006). Membranes were blocked using 3% fish gel in TBST as blocking reagent, followed by overnight incubation of the membrane with primary antibody in 5% BSA. The following primary antibodies were used: AMPK^Thr172^ phosphorylation (#2535S), mTOR protein (#2972), mTOR^Ser2448^ phosphorylation (#2971), Beclin1 protein (#3738), BNIP3 protein (#3769), DRP1 protein (#8570), Parkin protein (#4211), ULK^Ser757^ phosphorylation (#6888), ULK^Ser317^ phosphorylation (#12753), all from Cell Signaling Technologies (Danvers, MA), p62 (ab56416), (Abcam, Cambridge, UK), LC3I + LC3II protein (NB100‐2220), (Novus Biologicals, Littleton, CO) and AMPKα1 protein (G. Hardie, Dundee, UK). Membranes were incubated with appropriate horse radish peroxidase (HRP) conjugated secondary antibody (Dako, Glostrup, Denmark) (in 3% fish gel + TBST), developed with Luminata Classico or Forte Western HRP substrate (Millipore) and bands were visualized with a luminescent image analyzer (ImageQuant LAS 4000, GE Healthcare, Life Sciences). The results were quantified using ImageQuant TL software (GE Healthcare). Protein content of specific proteins was expressed as arbitrary units relative to pooled control samples loaded on each side of the gels. For each specific protein, a series of different standard loads were included to ensure that protein analyses were performed within the linear range. Phosphorylated proteins were normalized to the given total of that protein. No effect of genotype or intervention was observed for GAPDH protein, supporting equal protein concentration of all samples.

### Statistics

A two‐way analysis of variance (ANOVA) was applied to evaluate the effect of genotype and group as well as interaction on mRNA content, protein content, and phosphorylation level. Furthermore, a three‐way ANOVA was applied to test for effects of exercise training, acute exercise, and genotype (Fig. 3, 5B and Table 3). Student‐Newman‐Keuls post hoc test was used to locate significant differences when applicable. If the equal variance test failed, the data were logarithmically transformed before applying the ANOVA. Differences were considered significant at *P *< 0.05. All data are presented as mean ± standard error (SE). SigmaPlot 13.0 was used as statistical software (SYSTAT software Inc.).

**Figure 1:**
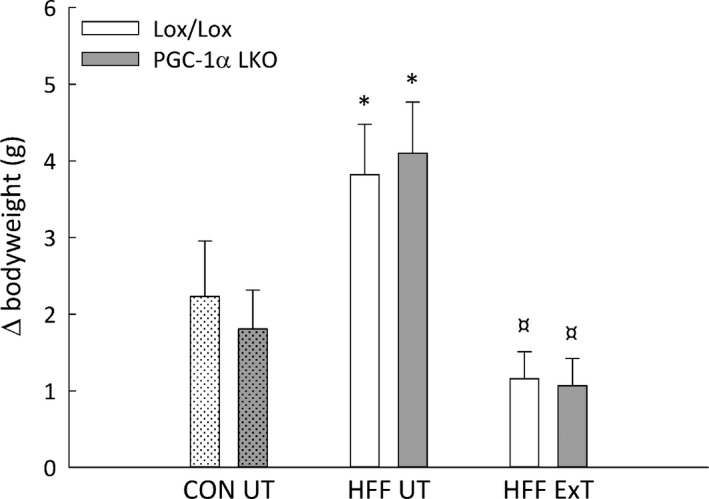
Body weight gain in liver‐specific PGC‐1α knockout (LKO) and littermate control (Lox/Lox) mice fed a control diet (CON) or a high‐fat high‐fructose diet (HFF) for 13 weeks. Half of the HFF mice performed treadmill exercise training the last 5 weeks (HFF ExT), the other half remained untrained (HFF UT). Values are presented as mean ± SE,* n *= 9–10. *: Significantly different from CON UT,* P *< 0.05. ¤: Significantly different from HFF UT,* P *< 0.05. Genotype *P *= 0.697; intervention *P *= 0.001; interaction *P *= 0.583.

**Figure 2:**
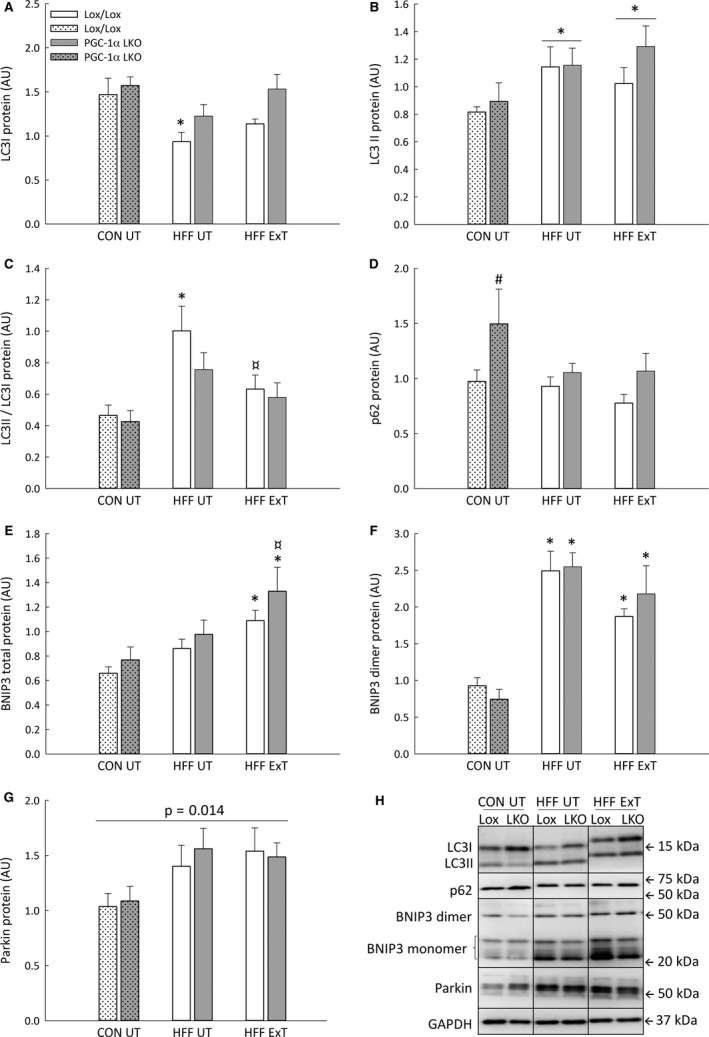
LC3I (A), LC3II (B), LC3II/LC3I protein ratio (C), p62 (D), BNIP3 total (E), BNIP3 dimer protein (F) and Parkin (G) protein content in liver from liver‐specific PGC‐1α knockout (LKO) and littermate control (Lox/Lox) mice fed a control diet (CON) or a high fat – high fructose diet (HFF) for 13 weeks. Half of the HFF mice performed treadmill exercise training the last 5 weeks (HFF ExT), the other half remained untrained (HFF UT). Representative blots for proteins A–G (H). Protein content is given as arbitrary units (AU). Values are presented as mean ± SE,* n *= 8–10. *: Significantly different from CON UT within given genotype, *P *< 0.05. ¤: Significantly different from HFF UT within given genotype, *P *< 0.05. #: Significantly different from Lox/Lox within given group, *P *< 0.05. A line designates a main effect, *P *< 0.05. Two‐way ANOVA: LC3I, intervention *P *= 0.003; genotype *P *= 0.011; interaction *P *= 0.662. LC3II, intervention *P *= 0.027; genotype *P *= 0.248; interaction *P *= 0.569. LC3II/LC3I, intervention *P *= 0.001; genotype *P *= 0.201; interaction *P *= 0.555. p62, intervention *P *= 0.148; genotype *P *= 0.025; interaction *P *= 0.486. BNIP3 total, intervention *P *< 0.001; genotype *P *= 0.100; interaction *P *= 0.814. BNIP3 dimer, intervention *P *< 0.001; genotype *P *= 0.443; interaction *P *= 0.405. Parkin, intervention *P *= 0.014; genotype *P *= 0.703; interaction *P *= 0.820. GAPDH, intervention *P *= 0.573; genotype *P *= 0.240; interaction *P *= 0.640.

**Table 2:** Effect of HFF and exercise training

	CON UT		HFF UT		HFF ExT	
	Lox/Lox	PGC‐1α LKO	Lox/Lox	PGC‐1α LKO	Lox/Lox	PGC‐1α LKO
DRP1 protein (AU)	2.2 ± 0.2	2.2 ± 0.2	2.1 ± 0.2	2.6 ± 0.3	2.2 ± 0.2	2.4 ± 0.2
Beclin1 protein (AU)	0.7 ± 0.0	0.7 ± 0.1	0.6 ± 0.1	0.7 ± 0.1	0.6 ± 0.1	0.7 ± 0.1
BNIP3 monomer protein (AU)	0.6 ± 0.1	0.8 ± 0.1	0.7 ± 0.1	0.8 ± 0.1	1.0 ± 0.1	1.3 ± 0.2^a^¤
GAPDH protein (AU)	1.1 ± 0.1	1.0 ± 0.0	1.1 ± 0.1	1.1 ± 0.1	1.0 ± 0.1	1.0 ± 0.1

DRP1, Beclin1, BNIP3 monomer, and GAPDH protein content in liver from liver‐specific PGC‐1α knockout (LKO) and littermate control (Lox/Lox) mice fed a control diet (CON) or a high‐fat high‐fructose diet (HFF) for 13 weeks. Half of the HFF mice performed treadmill exercise training the last 5 weeks (HFF ExT), the other half remained untrained (HFF UT). Values are presented as mean ± SE, *n *= 8–10.

a Significantly different from CON UT within given genotype, *P *< 0.05. ¤: Significantly different from HFF UT within given genotype, *P *< 0.05. Two‐way ANOVA: DRP1, intervention *P *= 0.908; genotype *P *= 0.803; interaction *P *= 0.430. Beclin1, intervention *P *= 0.411; genotype *P *= 0.575; interaction *P *= 0.961. BNIP3 monomer, intervention *P* < 0.001; genotype *P *= 0.143; interaction *P *= 0.636. GAPDH, intervention *P *= 0.573; genotype *P *= 0.240; interaction *P *= 0.640.

## Results

### Weight gain

The weight gain over 13 weeks was ~2 fold higher (*P *< 0.05) in Lox/Lox and PGC‐1α LKO mice receiving the HFF diet than mice receiving a control diet and exercise training prevented this increase. There was no difference in weight gain between the genotypes (Fig. 1).

**Figure 3:**
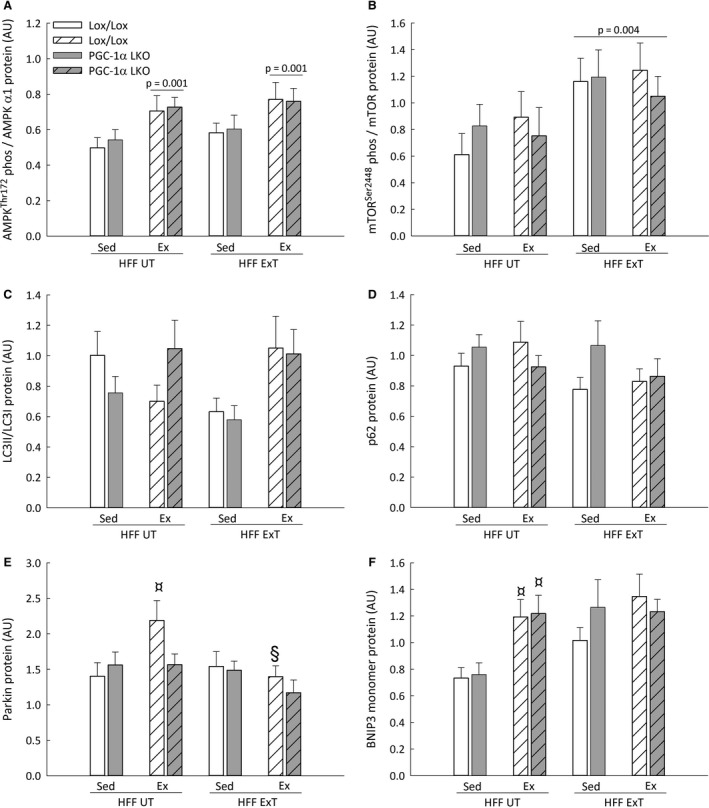
AMPK^T^ ^hr172^ phosphorylation normalized to AMPKα1 (A), mTOR^S^ ^er2448^ phosphorylation normalized to mTOR (B), LC3II/LC3I protein ratio (C), p62 (D), Parkin (E) and BNIP3 monomer (F) protein content in liver from liver‐specific PGC‐1α knockout (LKO) and littermate control (Lox/Lox) mice fed a high fat – high fructose diet (HFF) for 13 weeks. Half of the HFF mice performed treadmill exercise training the last 5 weeks (HFF ExT), the other half remained untrained (HFF UT). By the end of the 13 weeks, the two groups were again divided into sedentary (HFF UT Sed and HFF ExT Sed) groups and groups performing a 1 h acute running bout (HFF UT Ex and HFF ExT Ex). Protein content is given as arbitrary units (AU). Values are presented as mean ± SE,* n *= 8–10. ¤: Significantly different from HFF UT Sed within given genotype, *P *< 0.05. §: Significantly different from HFF UT Ex within given genotype, *P *< 0.05. A line and *P*‐value designates a main effect, *P *< 0.05. Two‐way ANOVA: AMPK^T^ ^hr172^ phosphorylation / AMPKα1, intervention *P *= 0.003; genotype *P *= 0.698; interaction *P *= 0.984. mTOR^S^ ^er2448^ phosphorylation / mTOR, intervention *P *= 0.034; genotype *P *= 0.869; interaction *P *= 0.679. LC3II/LC3I, intervention *P *= 0.141; genotype *P *= 0.817; interaction *P *= 0.534. p62, intervention *P *= 0.433; genotype *P *= 0.348; interaction *P *= 0.200. Parkin, intervention *P *= 0.029; genotype *P *= 0.187; interaction *P *= 0.228. BNIP3 monomer, intervention *P *< 0.001; genotype *P *= 0.617; interaction *P *= 0.599. GAPDH, intervention *P *= 0.401; genotype *P *= 0.459; interaction *P *= 0.908. Three‐way ANOVA: AMPK^T^ ^hr172^ phosphorylation / AMPKα1, Acute Ex *P *< 0.001; mTOR^S^ ^er2448^ phosphorylation / mTOR, ExT *P *= 0.004. Parkin, ExT x Acute Ex *P *= 0.028. BNIP3 monomer, ExT *P *= 0.013; Acute Ex *P *= 0.002.

### Liver weight and liver triglyceride content

Previously reported results from the same study as the present have shown that total body weight was higher (*P *< 0.05) in HFF UT mice than CON UT and HFF ExT mice in both genotypes (C. M. Kristensen, M. M. Dethlefsen, A. S. Tøndering, S. B. Lassen, J. N. Meldgaard, S. Ringholm, H. Pilegaard, unpublished data). Moreover, liver weight normalized to body weight was higher (*P *< 0.05) in HFF UT than CON UT in both genotypes and in Lox/Lox mice liver weight was higher (*P *< 0.05) in HFF ExT than CON UT and lower (*P *< 0.05) in HFF ExT than HFF UT. Hepatic triglyceride content was higher (*P *< 0.05) in HFF UT than CON UT mice in both genotypes and higher (*P *< 0.05) in HFF ExT than CON UT in Lox/Lox and LKO mice. In addition, the content of hepatic TG was lower (*P *< 0.05) in HFF ExT than HFF UT in LKO mice, but there was no difference in liver TG between genotypes in any of the groups (C. M. Kristensen, M. M. Dethlefsen, A. S. Tøndering, S. B. Lassen, J. N. Meldgaard, S. Ringholm, H. Pilegaard, unpublished data).

### Effect of HFF and exercise training on autophagy and mitophagy markers

Hepatic LC3I protein content was ~35% lower (*P *< 0.05) in HFF UT than CON UT in Lox/Lox mice, with no significant difference between HFF UT and HFF ExT and no effect on LC3I protein content in PGC‐1α LKO mice. There was no difference between genotypes in hepatic LC3I in any of the groups (Fig. 2A, H).

**Figure 4:**
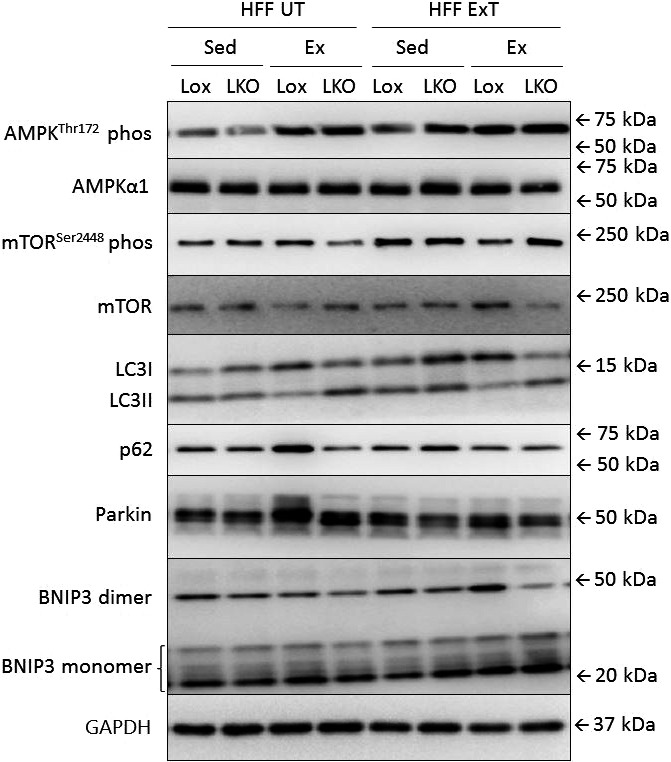
Representative blots of AMPK^T^ ^hr172^ phosphorylation, AMPKα1 protein, mTOR^S^ ^er2448^ phosphorylation, mTOR protein, LC3I protein, LC3II protein, p62 protein, Parkin protein, BNIP3 monomer and dimer protein in liver from liver‐specific PGC‐1α knockout (LKO) and littermate control (Lox/Lox) mice fed a control diet ( CON ) or a high fat – high fructose diet (HFF) for 13 weeks. Half of the HFF mice performed treadmill exercise training the last 5 weeks (HFF ExT), the other half remained untrained (HFF UT). By the end of the 13 weeks, the two groups were again divided into sedentary (HFF UT Sed and HFF ExT Sed) groups and groups performing a 1 h acute running bout (HFF UT Ex and HFF ExT Ex).

There was a main effect of diet on liver LC3II protein content and hepatic LC3II was ~1.5 fold higher (*P *< 0.05) in HFF UT and HFF ExT than CON UT. There was no difference between genotypes in any of the groups (Fig. 2B, H).

The LC3II/LC3I protein ratio was ~2 fold higher (*P *< 0.05) in HFF UT than CON UT in Lox/Lox mice, while there was no effect of diet and exercise training on LC3II/LC3I protein ratio in PGC‐1α LKO mice. There was no effect of genotype in any of the groups (Fig. 2C, H).

There was no effect of diet or exercise training on liver p62 protein content in either genotype, while p62 protein was ~1.5 fold higher (*P *< 0.05) in PGC‐1α LKO mice than Lox/Lox mice in the CON UT group (Fig. 2D, H).

Hepatic BNIP3 total protein content was ~1.5 fold higher (*P *< 0.05) in HFF ExT than CON UT in both genotypes, and ~1.7 fold higher (*P *< 0.05) in HFF ExT than HFF UT in PGC‐1α LKO mice. There was no difference between genotypes in any of the groups (Fig. 2E, H).

Hepatic BNIP3 monomer protein content was ~1.5 fold higher (*P *< 0.05) in HFF ExT than CON UT and HFF UT in PGC‐1α LKO mice and there was no difference between genotypes in any of the groups (Table 2). BNIP3 dimer protein was ~2–3.6 fold higher (*P *< 0.05) in HFF UT and HFF ExT than CON UT in both genotypes, with no difference between genotypes in any of the groups (Fig. 2F, H).

There was a main effect of the intervention in hepatic Parkin protein content, with ~1.5 fold higher (*P *< 0.05) Parkin protein in the HFF UT and HFF ExT groups than the CON UT group in both genotypes. There was no difference between genotypes in any of the groups (Fig. 2G, H).

There was no effect of HFF diet, exercise training or genotype on hepatic Dynamin‐1‐like protein (DRP1), Beclin1, and GAPDH protein content (Table 2).

Representative blots for proteins in Table 2 are shown in Figure 6.

**Figure 5:**
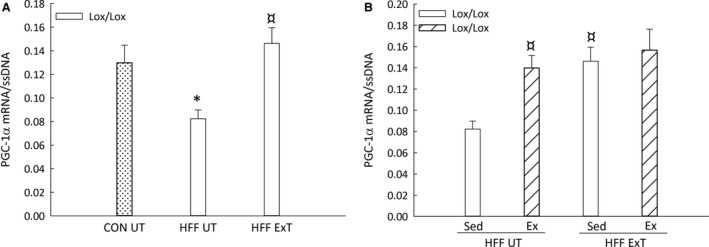
PGC‐1α mRNA content in liver from liver‐specific PGC‐1α knockout (LKO) and littermate control (Lox/Lox) mice fed a control diet (CON) or a high‐fat high‐fructose diet (HFF) for 13 weeks. Half of the HFF mice performed treadmill exercise training the last 5 weeks (HFF ExT), the other half remained untrained (HFF UT) (A). By the end of the 13 weeks, the two groups were again divided into sedentary (HFF UT Sed and HFF ExT Sed) groups and groups performing a 1 h acute running bout (HFF UT Ex and HFF ExT Ex) (B). mRNA is normalized to single‐stranded (ss) DNA. Values are presented as mean ± SE,* n *= 8–10. *Significantly different from CON UT,* P *< 0.05. ¤: Significantly different from HFF UT,* P *< 0.05. Two‐way ANOVA: PGC‐1α mRNA (A), HFF *P *= 0.015; ExT *P *= 0.002. PGC‐1α mRNA (B), ExT x Acute Ex *P *= 0.021.

### Effect of acute exercise on autophagy and mitophagy markers

Hepatic AMPK^Thr172^ phosphorylation normalized to AMPKα1 protein content was ~1.5 fold higher (*P *< 0.05) in HFF UT Ex and HFF ExT Ex than HFF UT Sed and HFF ExT Sed, respectively, in both genotypes. There was no effect of genotype on AMPK^Thr172^ phosphorylation normalized to AMPKα1 protein (Fig. 3A). There was a main effect (*P *< 0.05) of acute exercise in hepatic AMPKα1 protein content in both genotypes, while there was no difference between genotypes (Table 3).

**Table 3:** Effect of Exercise training and acute exercise

	**HFF UT Sed**		**HFF UT Ex**		**HFF ExT Sed**		**HFF ExT Ex**	
	**Lox/Lox**	**PGC‐1α LKO**	**Lox/Lox**	**PGC‐1α LKO**	**Lox/Lox**	**PGC‐1α LKO**	**Lox/Lox**	**PGC‐1α LKO**
AMPKα1 protein (AU)	0.5 ± 0.1	0.5 ± 0.1	0.7 ± 0.1	0.7 ± 0.1	0.6 ± 0.1	0.6 ± 0.1	0.8 ± 0.1	0.8 ± 0.1 c
mTOR protein (AU)	1.2 ± 0.1	1.2 ± 0.1	1.2 ± 0.2	1.2 ± 0.1	0.9 ± 0.1	0.8 ± 0.1	1.0 ± 0.1	0.9 ± 0.2 d
ULK^Ser317^ phos/ULK1 protein (AU)	3.0 ± 0.4	2.7 ± 0.4	2.9 ± 0.3	2.7 ± 0.4	2.1 ± 0.2	2.7 ± 0.3	3.2 ± 0.4	2.7 ± 0.4
ULK^Ser757^ phos/ULK1 protein (AU)	1.3 ± 0.3	1.0 ± 0.2	0.8 ± 0.1	0.8 ± 0.1	1.0 ± 0.2	0.8 ± 0.1	0.8 ± 0.2	0.7 ± 0.1
ULK1 protein (AU)	0.6 ± 0.1	0.6 ± 0.1	0.6 ± 0.1	0.6 ± 0.1	0.5 ± 0.1	0.5 ± 0.0	0.4 ± 0.0	0.5 ± 0.1 d
LC3I protein (AU)	0.9 ± 0.1	1.2 ± 0.1	1.2 ± 0.1	0.9 ± 0.1	1.1 ± 0.1	1.5 ± 0.2^b^	1.1 ± 0.2	0.9 ± 0.1 a ^,^ e
LC3II protein (AU)	1.1 ± 0.1	1.2 ± 0.1	1.2 ± 0.1	1.4 ± 0.2	1.0 ± 0.1	1.3 ± 0.1	1.2 ± 0.2	1.2 ± 0.1
BNIP3 total protein (AU)	0.9 ± 0.1	1.0 ± 0.1	1.1 ± 0.1	1.2 ± 0.1	1.1 ± 0.1	1.3 ± 0.2	1.4 ± 0.2	1.3 ± 0.1 d
BNIP3 dimer protein (AU)	2.5 ± 0.3	2.5 ± 0.2	1.8 ± 0.3	2.4 ± 0.4	1.9 ± 0.1	2.2 ± 0.4	1.7 ± 0.3	1.4 ± 0.2 d, c
DRP1 protein (AU)	2.1 ± 0.2	2.6 ± 0.3	2.3 ± 0.2	2.1 ± 0.2	2.2 ± 0.2	2.4 ± 0.2	2.6 ± 0.3	2.1 ± 0.1 d, c
Beclin1 protein (AU)	0.6 ± 0.1	0.7 ± 0.1	0.8 ± 0.1	0.8 ± 0.1	0.6 ± 0.1	0.7 ± 0.1	0.6 ± 0.1	0.6 ± 0.1
GAPDH protein (AU)	1.1 ± 0.1	1.1 ± 0.1	1.0 ± 0.1	1.0 ± 0.1	1.0 ± 0.1	1.0 ± 0.1	1.0 ± 0.1	1.0 ± 0.1

AMPKα1, mTOR, ULK^Ser317^ phosphorylation/ULK1, ULK^Ser757^ phosphorylation/ULK1, ULK1, LC3I, LC3II, BNIP3 total, BNIP3 dimer, DRP1, Beclin1, and GAPDH protein content in liver from liver specific PGC‐1α knockout (LKO) and littermate control (Lox/Lox) mice fed a high‐fat high‐fructose diet for 13 weeks. Half of the mice performed treadmill exercise training the last 5 weeks (HFF ExT), the other half remained untrained (HFF UT). By the end of the 13 weeks, the two groups were again divided into sedentary (HFF UT Sed and HFF ExT Sed) groups and groups performing a 1 h acute running bout (HFF UT Ex and HFF ExT Ex). Protein content is given as arbitrary units (AU). Values are presented as mean ± SE, *n *= 8–10.

a Significantly different from HFF ExT Sed within given genotype, *P *< 0.05.

b Significantly different from Lox/Lox within given group, *P *< 0.05.

c Main effect of acute exercise, *P *< 0.05.

d Main effect of exercise training, *P *< 0.05.

e Interaction: the acute exercise effect depended on the genotype, *P *< 0.05.

Two‐way ANOVA: AMPKα1, intervention *P *= 0.931; genotype *P *= 0.663; interaction *P *= 0.311. mTOR, intervention *P *= 0.028; genotype *P *= 0.376; interaction *P *= 0.964. ULK^Ser317^ phosphorylation / ULK1, intervention *P *= 0.401; genotype *P *= 0.774; interaction *P *= 0.396. ULK^Ser757^ phosphorylation / ULK1, intervention *P *= 0.605; genotype *P *= 0.318; interaction *P *= 0.839. ULK1, intervention *P *= 0.141; genotype *P *= 0.342; interaction *P *= 0.580. LC3I, interaction *P *= 0.015. LC3II, intervention *P *= 0.736; genotype *P *= 0.323; interaction *P *= 0.636. BNIP3 total, intervention *P *= 0.011; genotype *P *= 0.411; interaction *P *= 0.526. BNIP3 dimer, intervention *P *= 0.019; genotype *P *= 0.355; interaction *P *= 0.488. DRP1, intervention *P *= 0.805; genotype *P *= 0.911; interaction *P *= 0.088. Beclin1, intervention *P* = 0.194; genotype *P* = 0.558; interaction *P* = 0.838. GAPDH, intervention *P* = 0.401; genotype *P* = 0.459; interaction *P* = 0.908. Three‐way ANOVA: AMPKα1, Acute Ex *P *< 0.001. mTOR, ExT *P* = 0.003. ULK1, ExT *P* = 0.031. LC3I, ExT x Acute Ex *P* = 0.019; Acute Ex x geno *P* = 0.001. BNIP3 total, ExT *P* = 0.014. BNIP3 dimer, ExT *P* = 0.012; Acute Ex *P* = 0.025. DRP1, Acute Ex x geno *P* = 0.009.

Hepatic mTOR^Ser2448^ phosphorylation normalized to total mTOR protein content was ~1.5‐fold higher (*P *< 0.05) in HFF ExT Sed and HFF ExT Ex than HFF UT Sed and HFF UT Ex in both genotypes. There was no effect of acute exercise on mTOR^Ser2448^ phosphorylation normalized to total mTOR protein content in either genotype (Fig. 3B). There was a main effect (*P *< 0.05) of exercise training in hepatic total mTOR protein content, while there was no difference between genotypes (Table 3).

There was no effect of acute exercise on ULK^Ser317^ and ULK^Ser757^ phosphorylation normalized to ULK1 in either genotype and there was no difference between genotypes in ULK^Ser317^ and ULK^Ser757^ phosphorylation normalized to ULK1 protein in any of the groups (Table 3). There was a main effect (*P *< 0.05) of exercise training in hepatic ULK1 protein content, while there was no difference between genotypes (Table 3).

There was a main intervention effect (*P *< 0.05) in hepatic LC3I protein content, where the acute exercise effect depended on the genotype. Moreover, hepatic LC3I protein content was in HFF ExT ~40% lower (*P *< 0.05) after acute exercise than in sedentary in PGC‐1α LKO mice, while there was no effect of acute exercise in Lox/Lox mice. LC3I protein was ~1.4‐fold higher (*P *< 0.05) in LKO than Lox/Lox mice in the HFF ExT Sed group (Table 3).

There was no effect of acute exercise in either genotype and no effect of genotype on hepatic LC3II protein content in any of the groups (Table 3).

There was no effect of acute exercise in either genotype and no effect of genotype on hepatic LC3II/LC3I protein ratio or p62 protein content (Fig. 3C and D).

Hepatic Parkin protein content was ~1.5 fold higher (*P *< 0.05) in HFF UT Ex than HFF UT Sed in Lox/Lox mice, but with no effect of acute exercise in HFF ExT in Lox/Lox mice, and there was no effect of acute exercise in PGC‐1α LKO mice and no difference between genotypes (Fig. 3E).

Hepatic BNIP3 monomer protein was ~1.6 fold higher (*P *< 0.05) in HFF UT Ex than HFF UT Sed Lox/Lox and LKO mice. There was no difference between genotypes in BNIP3 monomer protein content (Fig. 3F).

There was a main effect (*P *< 0.05) of exercise training in total BNIP3 and BNIP3 dimer protein content and a main effect (*P *< 0.05) of acute exercise in BNIP3 dimer protein content in both genotypes. There was no difference in BNIP3 total or BNIP3 dimer protein content between genotypes (Table 3).

There was no effect of exercise training or acute exercise on hepatic DRP1, Beclin1, and GAPDH protein content in either genotype and no difference between genotypes in any of the groups (Table 3).

Representative blots for proteins given in Figure 3 are given in Figure 4. Representative blots given in Table 3 are shown in Figure 7.

**Figure 6:**
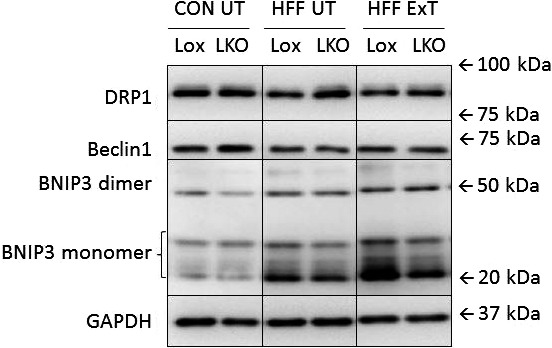
Representative blots of DRP1 protein, Beclin1 protein, BNIP3 monomer and dimer protein and GAPDH protein in liver from liver‐specific PGC‐1α knockout (LKO) and littermate control (Lox/Lox) mice fed a control diet (CON) or a high‐fat high‐fructose (HFF) for 13 weeks. Half of the HFF mice performed treadmill exercise training the last 5 weeks (HFF ExT), the other half remained untrained (HFF UT).

### PGC‐1α mRNA

Hepatic PGC‐1α mRNA content was ~40% lower (*P *< 0.05) in HFF UT than in CON UT mice and ~1.8 fold higher (*P *< 0.05) in HFF ExT than the HFF UT in Lox/Lox mice (Fig. 5A). Hepatic PGC‐1α mRNA was in HFF UT ~1.7 fold higher (*P *< 0.05) after acute exercise than at rest, while there was no difference between acutely exercised and sedentary Lox/Lox mice in the HFF ExT group (Fig. 5B).

**Figure 7:**
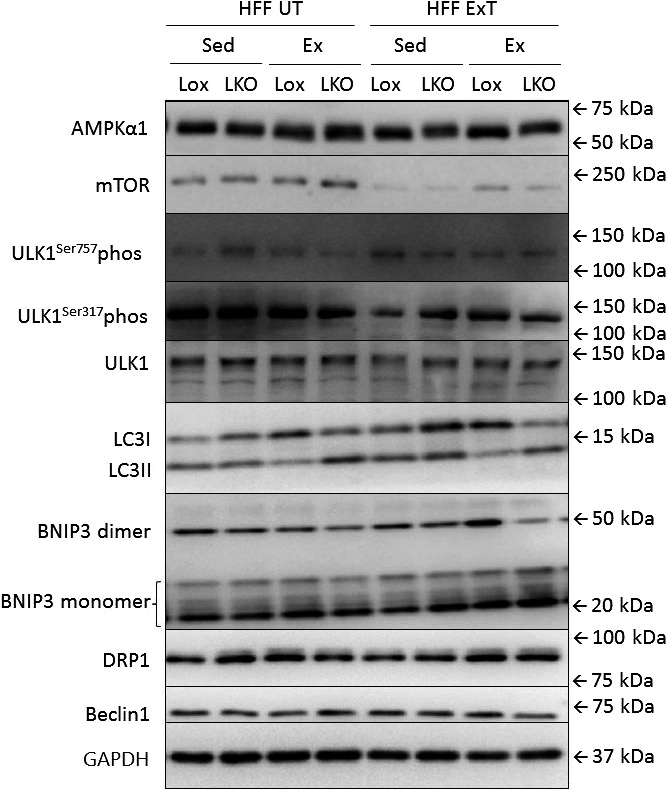
Representative blots of AMPKα1 protein, mTOR protein, DRP1 protein, ULK^S^ ^er317^ phosphorylation, ULK^S^ ^er757^ phosphorylation, ULK1 protein, LC3I protein, LC3II protein, BNIP3 monomer and dimer protein, Beclin1 protein and GAPDH protein in liver from liver specific PGC‐1α knockout (LKO) and littermate control (Lox/Lox) mice fed a high‐fat high‐fructose diet for 13 weeks. Half of the mice performed treadmill exercise training the last 5 weeks (HFF ExT), the other half remained untrained (HFF UT). By the end of the 13 weeks, the two groups were again divided into sedentary (HFF UT Sed and HFF ExT Sed) groups and groups performing a 1 h acute running bout (HFF UT Ex and HFF ExT Ex).

## Discussion

The main findings of this study are that hig‐fat high‐fructose (HFF) diet increased hepatic Parkin and BNIP3 dimer protein, with no effect of exercise training on Parkin protein. In addition, exercise training reversed the HFF‐induced increase in LC3II/LC3I protein ratio, but with no change in LC3II and p62 protein. Furthermore, exercise training rescued a diet‐induced reduction in hepatic PGC‐1α mRNA and acute exercise increased PGC‐1α mRNA in HFF‐fed untrained mice, but not in HFF‐fed exercise trained mice, at 2h of recovery.

The present finding that LC3II/LC3I protein ratio increased and LC3I decreased in the liver with HFF is corroborated by several other studies reporting increased LC3II/LC3I protein ratios in the liver with HFD feeding (Gonzalez‐Rodriguez *et al*. 2014, Hsu *et al*. 2016, Wang *et al*. 2017a). This may imply that more LC3I was converted to LC3II with concomitant increased autophagosome number and autophagy with HFF feeding. On the other hand, the lack of change in LC3II protein levels with HFF, may suggest that the decreased LC3I protein level rather reflects reduced capacity for autophagy regulation, as previously suggested (Gonzalez‐Rodriguez *et al*. 2014). This possibility is in agreement with some previous studies observing increased p62 protein in the liver with HFD feeding (Gonzalez‐Rodriguez *et al*. 2014, Liu *et al*. 2015, Tanaka *et al*. 2016, Wang *et al*. 2017a, Wang *et al*. 2017b), but not supported by the present observation that p62 protein was unchanged with HFF diet. Nevertheless, the lack of change in hepatic p62 protein is in accordance with previous mouse studies using sucrose supplement (Alex *et al*. 2015) and HFD (Rosa‐Caldwell *et al*. 2017) and does not support increased autophagy in the liver in response to HFF.

The novel observation in the present study that hepatic Parkin and BNIP3 dimer protein increased with HFF may be interpreted as increased capacity for mitophagy. Mitophagy relies on removal of damaged mitochondria through autophagosomal degradation, which did not appear to be increased with HFF in the present study, based on the lack of change in LC3II and p62 protein. A key aspect in maintaining mitochondrial function is removing damaged mitochondria through mitophagy (Pickles *et al*. 2018), and the increased Parkin and BNIP3 dimer protein may therefore be interpreted as an adaptation to an accumulation of dysfunctional mitochondria, as previously suggested for Parkin (Narendra *et al*. 2008) and indicated for BNIP3 (Chen *et al*. 1997, Gustafsson 2011, Sowter *et al*. 2001). BNIP3 dimerization is necessary for incorporation of the protein into the mitochondrial outer membrane (Bocharov *et al*. 2007) and recombinant dimerized BNIP3 has been shown to increase opening of mitochondrial permeability transition pores (mPTP) (Zhang & Ney 2009). This has been suggested to serve as a mitochondrial valve, allowing release of built up reactive oxygen species (ROS) and lipid byproducts (Kwong & Molkentin 2015) and in accordance, BNIP3 has been shown to cause opening of the mPTP and trigger apoptosis through cytochrome c release in isolated liver mitochondria (Kim *et al*. 2002). Because apoptosis has been reported in the liver of HFD fed rats (Wang *et al*. 2008), it may be speculated that the increased dimerization of BNIP3 with HFF in the present study reflects built up ROS and lipid or increased BNIP3‐mediated cell death. Taken together, hepatic autophagy seemed unchanged with HFF with potentially decreased capacity for autophagy and increased dimerization of BNIP3 and Parkin protein content, which may indicate accumulation of dysfunctional mitochondria or that other degradative pathways become activated, but this remains to be determined.

The present findings that exercise training decreased body weight gain and liver weight as well as increased glucose tolerance in HFF‐fed mice as reported previously (C. M. Kristensen, M. M. Dethlefsen, A. S. Tøndering, S. B. Lassen, J. N. Meldgaard, S. Ringholm, H. Pilegaard, unpublished data), are corroborated by studies reporting several benefits of exercise training in HFD‐fed mice (Alex *et al*. 2015, Kawanishi *et al*. 2012). However, the finding that there was no change in LC3I, LC3II or p62 protein levels with exercise training compared with either untrained CON or HFF mice indicates that exercise training did not induce hepatic autophagy. This is not in line with previous studies, reporting increased LC3II/LC3I protein ratio and decreased p62 protein in the liver of exercise trained HFD‐fed mice (Rosa‐Caldwell *et al*. 2017, Wang *et al*. 2017a). On the other hand, the observation that there was no difference in the LC3II/LC3I ratio between untrained mice on regular diet and exercise trained mice on HFF may suggest that exercise training reversed the effect of HFF on autophagy. The differences between the previous and the present study may be explained by the study designs, because the mice in one study (Rosa‐Caldwell *et al*. 2017) exercise trained for the entire duration of the diet intervention and in another (Wang *et al*. 2017a) used voluntary wheel running, while the present study used treadmill exercise training, which was initiated after 8 weeks of HFF feeding. The different findings may also be due to differences in diet composition, as hepatic autophagy was not increased in the liver of exercise trained mice receiving sucrose supplementation in the drinking water (Alex *et al*. 2015). The present observation that Parkin and BNIP3 dimer protein remained increased in exercise trained HFF‐fed mice, while total BNIP3 protein levels were higher in exercise trained HFF mice than in sedentary HFF mice, may indicate a further elevation in the capacity for mitophagy with exercise training in the present study. Taken together, exercise training did not seem to affect hepatic autophagy, but increased the capacity for mitophagy in HFF‐fed mice through enhanced total BNIP3, although the apparent lack of autophagy may indicate that exercise training did not change the capacity for removing dysfunctional mitochondria.

The finding that an acute bout of exercise increased the phosphorylation of AMPK^Thr172^ in the liver of both sedentary and exercise trained HFF mice is in accordance with previous studies on skeletal muscle (Leick *et al*. 2008, Winder & Hardie 1996) and possibly liver (Ghareghani *et al*. 2017). This indicates that the mice in the present study were similarly metabolically challenged by the acute exercise bout, when the untrained and trained mice exercised at the same absolute intensity. In addition, the increase in AMPK phosphorylation may suggest pro‐autophagy signaling, but the AMPK downstream target ULK^Ser317^ phosphorylation (Kim *et al*. 2011) did not reflect AMPK phosphorylation, suggesting that there was no acute hepatic autophagy signaling in the HFF‐fed mice. Moreover, the absence of activating or inhibitory phosphorylation of ULK1 is in accordance with the lack of change in any of the autophagic markers LC3I, LC3II, and p62 with acute exercise. The novel observations that hepatic BNIP3 monomers and Parkin protein increased with acute exercise in untrained mice suggest a rapid change in the capacity to regulate mitophagy. Moreover, the elevated basal level of BNIP3 monomer protein in exercise trained mice with no further increase with acute exercise may indicate that exercise trained mice were not sufficiently challenged by acute running at the same absolute exercise intensity as the untrained mice. This is supported by the novel finding that Parkin protein levels were not either increased with acute exercise in exercise trained HFF fed mice, as observed in HFF untrained mice. Together, these findings suggest that acute exercise enhanced the capacity for mitophagy regulation in untrained mice, and exercise training blunted this effect when exercising at the same absolute intensity in untrained and trained mice. Moreover, autophagy was unaffected by acute exercise in the liver of HFF‐fed mice independent of training state. This may suggest that acute exercise in HFF‐fed mice resulted in mitochondrial damage and hence increased the capacity for removal of mitochondria, while exercise trained mice were protected from exercise‐induced mitochondrial damage.

The finding that hepatic PGC‐1α mRNA decreased with HFF feeding is supported by other studies (Barroso *et al*. 2011, Barroso *et al*. 2017) and may indicate decreased hepatic mitochondrial biogenesis. Furthermore, the finding that exercise training rescued the HFF‐induced decrease in PGC‐1α mRNA is novel, but in accordance with observations in liver from exercise trained chow‐fed rodents (E L et al. 2013, Santos‐Alves *et al*. 2014). In addition, the impact of exercise training on hepatic PGC‐1α mRNA may suggest that the PGC‐1α‐mediated regulation of mitochondrial biogenesis (Handschin *et al*. 2007, Lira *et al*. 2010) and mitochondrial quality (Greene *et al*. 2015) was re‐established by exercise training despite of the HFF diet. The novel finding that hepatic PGC‐1α mRNA increased with acute exercise in HFF‐fed untrained mice, but not in the HFF‐fed exercise trained mice supports, that the exercise trained mice had obtained adaptations resulting in less metabolic challenge when exercising at the same absolute intensity as untrained mice. Of notice is that the exercise training did not affect OXPHOS protein in the liver indicating that exercise training did not affect the oxidative capacity of the liver. Moreover, previous studies reported a requirement for PGC‐1α in acute exercise and exercise training‐induced regulation of LC3II and LC3I in mouse skeletal muscle from chow‐fed mice (Brandt *et al*. 2017, Brandt *et al*. 2018, Halling *et al*. 2016, Vainshtein *et al*. 2015). Thus, the lack of difference between Lox/Lox and PGC‐1α LKO mice in any of the autophagy markers in this study indicates tissue‐specific PGC‐1α dependency in autophagy and mitophagy regulation.

In conclusion, this study indicates that a high‐fat high‐fructose diet decreased the capacity for regulation of autophagy and increased the capacity for regulation of mitophagy in the liver. Exercise training did not regulate autophagy, but enhanced the hepatic capacity for regulation of mitophagy in HFF mice. In addition, the present findings suggest that exercise training blunted acute exercise‐induced hepatic autophagy in mice on HFF diet. In addition, liver PGC‐1α had only minor effects on basal as well as exercise and exercise training‐mediated regulation of hepatic autophagy and mitophagy. Together, this suggests that hepatic autophagy and mitophagy are modulated by diet, exercise, and exercise training in a PGC‐1α‐independent manner.

## Conflicts of Interest

No conflicts of interest, financial or otherwise, are declared by the authors.
